# The role of spontaneous neurotransmission in synapse and circuit development

**DOI:** 10.1002/jnr.24154

**Published:** 2017-10-16

**Authors:** Laura C. Andreae, Juan Burrone

**Affiliations:** ^1^ Centre for Developmental Neurobiology, King's College London New Hunt's House, 4th Floor, Guy's Hospital Campus, London SE1 1UL UK; ^2^ MRC Centre for Neurodevelopmental Disorders, King's College London New Hunt's House, 4th Floor, Guy's Hospital Campus, London SE1 1UL UK; ^3^ FENS‐Kavli Network of Excellence, Europe‐wide

**Keywords:** spontaneous, miniature, neurotransmitter release, synapse formation, development, dendritic arbor

## Abstract

In the past, the spontaneous release of neurotransmitter from presynaptic terminals has been thought of as a side effect of evoked release, with little functional significance. As our understanding of the process of spontaneous release has increased over time, this notion has gradually changed. In this review, we focus on the importance of this form of release during neuronal development, a time of extreme levels of plasticity that includes the growth of dendrites and axons as well as the formation of new synaptic contacts. This period also encompasses high levels of neurotransmitter release from growing axons, and recent studies have found that spontaneous transmitter release plays an important role in shaping neuronal morphology as well as modulating the properties of newly forming synaptic contacts in the brain. Here, we bring together the latest findings across different species to argue that the spontaneous release of neurotransmitter is an important player in the wiring of the brain during development.

Communication between neurons largely happens at the chemical synapse, where neurotransmitters are released from presynaptic vesicles, cross the narrow synaptic cleft, and activate receptors on the postsynaptic neuron. The trigger for neurotransmitter release is the firing of an action potential in the presynaptic neuron; when depolarization invades the presynaptic terminal, voltage‐gated calcium channels open, and the rise in intracellular calcium at the terminal (or bouton) leads to fusion of vesicles with the plasma membrane and exocytosis of their contents. However, in addition to this activity‐dependent (or evoked) transmitter release, synaptic vesicles can spontaneously fuse and release a single packet (or quantum) of neurotransmitter. This spontaneous vesicular release can be detected postsynaptically as a miniature postsynaptic current. The discovery of this form of release by Bernard Katz in 1952 (Fatt & Katz, 1952) was fundamental to our understanding of the quantal nature of neurotransmission; however, spontaneous release itself was often regarded as ‘noise’ in the system, having no intrinsic function. Increasingly this view is now being challenged, and indeed recent work has demonstrated that the antidepressant actions of ketamine may act via effects on spontaneous neurotransmission, with important implications for developing new treatments for depression (Autry et al., 2011; Kavalali & Monteggia, 2015). In this review, we will focus on the role of spontaneous transmission during neuronal development.

In order to form a synapse, the presynaptic axon must make contact with the postsynaptic dendrite, following which, pre‐ and post‐synaptic machinery needs to be assembled at this site. As one might expect, this requires a host of guidance cues that aid the axon in finding its target and a variety of cell‐adhesion molecules that promote synapse formation (Sanes & Yamagata, 2009; Shen & Scheiffele, 2010). At the same time, axonal and dendritic arborizations progressively enlarge and become more complex, allowing more synapses to form and circuits to be built. These processes are clearly linked, and indeed may depend, to some extent, on each other (Vaughn, 1989). Traditionally, the role of neuronal activity has been seen as one of refinement, such that once the basic structure of connections is established, the less active ones can be pruned away, often via competitive mechanisms (Huberman, Feller, & Chapman, 2008; Katz & Shatz, 1996; LeVay, Wiesel, Hubel, 1980; Sanes & Lichtman, 1999). More recently, it is becoming clear that activity may also be important for synapse and circuit formation per se (Andreae & Burrone, 2014; Choi et al., 2014; Okawa et al., 2014; Sabo, Gomes, & McAllister, 2006), although this remains a controversial field (Sigler et al., 2017). Indeed, it seems that specifically spontaneous neurotransmission may regulate various aspects of this process.

## 1 NEUROTRANSMITTER RELEASE DURING DEVELOPMENT

It has long been known that immature neurons can release neurotransmitter from their axons before making contact with a postsynaptic neuron. Studies in cultured *Xenopus* spinal neurons found that both growth cones (Young & Poo, 1983) and sites along the axon (Dai & Peng, 1996; Zakharenko, Chang, O'Donoghue, & Popov, 1999) spontaneously released the neurotransmitter acetylcholine. Similarly, synaptic vesicles from both chick peripheral neurons (Hume, Role, & Fischbach, 1983; Tojima et al., 2007) and rodent central nervous system (CNS) neurons were also found to be capable of cycling before synapse formation (Kraszewski et al., 1995; Matteoli, Takei, Perin, Sudhof, & De Camilli, 1992; Sabo et al., 2006). While in some preparations, activity dependent release from these immature axons has been clearly demonstrated, such as *Xenopus* spinal neurons (Zakharenko et al., 1999), there were early indications that evoked release in mammalian CNS neurons may be developmentally regulated. Using the styryl dye FM2‐10 to label cycling vesicles, Mozhayeva et al. (2002) found that levels of evoked release in cultured rat hippocampal neurons progressively increased during development, and at the earliest stage studied (5DIV) almost no response to activity was seen.

But what of spontaneous neurotransmitter release in developing neurons? Spontaneous release had traditionally been studied by using whole‐cell patch clamp electrophysiology to record miniature postsynaptic currents (or ‘minis’) from the postsynaptic cell, and using this approach, a progressive increase in the frequency of these currents during development had been observed (Desai, Cudmore, Nelson, & Turrigiano, 2002; Mozhayeva, Sara, Liu, & Kavalali, 2002). However, this presumably reflects the known increase in synaptic connections, and restricts analysis to after synapse formation. To examine levels of spontaneous release directly at individual boutons, fluorescent reporters of vesicle exocytosis have been used to integrate the total number of release events over long periods of time, in the absence of any activity. One such reporter of presynaptic vesicle exocytosis, biosyn (a biotinylated VAMP2), is well suited to independently report both evoked and spontaneous vesicle cycling within the same presynaptic bouton, with different colors (Fredj & Burrone, 2009). Using this tool in cultured rat hippocampal neurons evoked release was found to increase with development in a manner similar to that previously observed (Mozhayeva et al., 2002), but spontaneous vesicle cycling was found to occur at exceptionally high levels in young neurons, which progressively decreased with maturation (Andreae, Fredj, & Burrone, 2012). Further analysis, including the additional use of the pH‐sensitive reporter synaptopHluorin, a transient reporter of vesicle exocytosis and endocytosis, indicated that at early stages before synapse formation (4‐5 days in vitro, DIV) evoked release was completely absent, despite robust calcium responses to depolarization. In agreement with findings in adult neurons, spontaneously cycling vesicles throughout development were found to derive from a vesicle pool which was independent of the vesicle pool cycling in response to activity, even though they coexisted in the same presynaptic bouton. Although circumstantial, the fact that developing mammalian CNS neurons exhibit very high levels of spontaneous synaptic vesicle cycling, in the absence of evoked release early on, suggests that spontaneous release has an important role to play during development.

## 2 SPONTANEOUS RELEASE REGULATES PRESYNAPTIC MATURATION

Evidence implicating spontaneous release in the maturation of presynaptic boutons comes from studies of the *Drosophila* neuromuscular junction (NMJ). An early clue emerged from work on *Drosophila* mutant flies that were null for the SNARE complex binding protein, complexin. These mutant flies exhibited an increase in spontaneous vesicle fusion at the NMJ, as well as an increased number of presynaptic boutons in the innervating motor neurons (Huntwork & Littleton, 2007). Although at this stage there was no evidence that one led to the other, in 2015 the Littleton group revisited this issue when they noticed that a number of different mutations that all resulted in increased spontaneous release at the NMJ (as identified by increased frequency of minis) were also all associated with increased numbers of presynaptic boutons. When they quantified the relative levels of these two features in the different mutants, they found that there was a direct correlation between the level of increase in spontaneous release and the number of boutons (Cho et al., 2015). Detailed characterization of this phenomenon led them to identify postsynaptic syt4 and presynaptic BMP release as critical for the increase in presynaptic growth.

**Figure 1:**
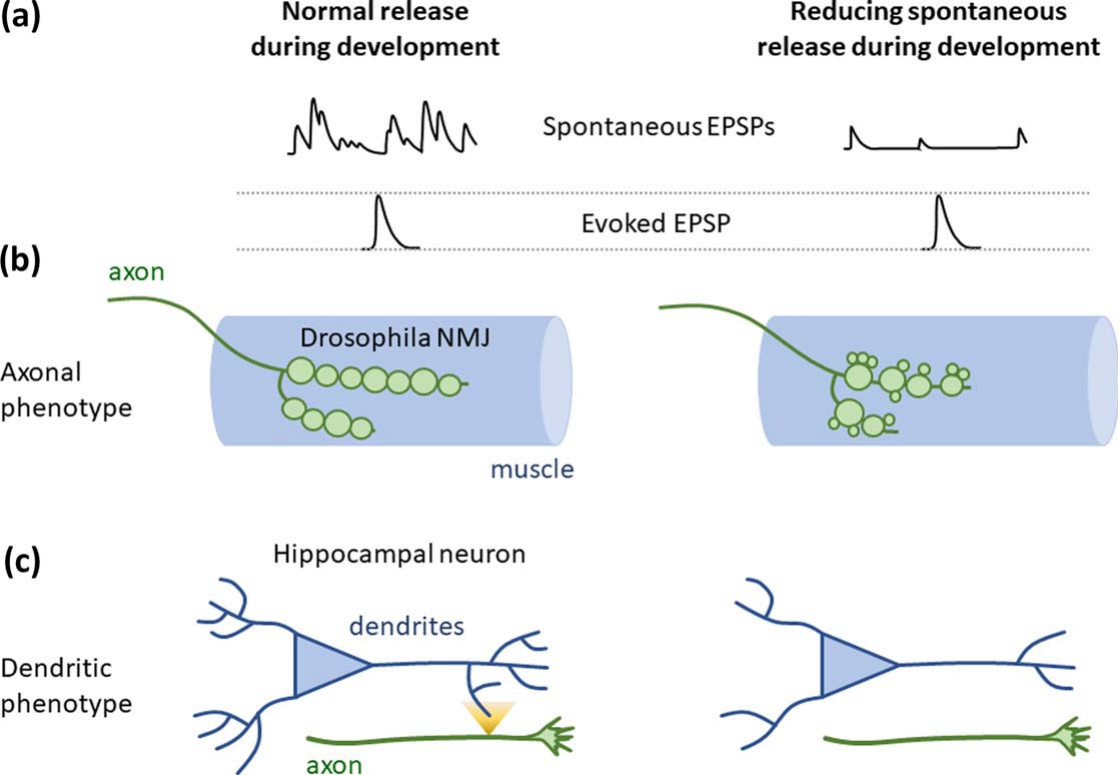
Spontaneous release is important for axonal and dendritic development. **A**. Neurons undergo both spontaneous and evoked release during development (traces on the left) and tools can be used to selectively interfere with spontaneous release (traces on the right). **B**. At the *Drosophila* neuromuscular junction (NMJ), interfering with spontaneous neurotransmitter release, but not evoked release, during the period of synaptogenesis causes the formation of aberrant presynaptic terminal boutons. These are mainly observed as an increased number of small presynaptic boutons and a decrease in overall synaptic terminal area. **C**. In mammalian hippocampal neurons, where release from developing axons occurs predominantly in a spontaneous manner and activates dendritic NMDA receptors (yellow event), interfering with NMDAR activity driven by spontaneous release results in less complex dendritic arbors

In line with these findings and lending further support to an instructive role for spontaneous release during development, another key study in the *Drosophila* NMJ carefully dissected out the role of spontaneous neurotransmission on the maturation of synaptic boutons themselves (Choi et al., 2014; Figure 1). The study found that specifically blocking spontaneous release resulted in an increased proportion of small presynaptic boutons and a generally reduced motor neuron terminal area, which could not be rescued by increasing evoked release (Figure 1B). The effect on boutons was due to a slowing down of bouton enlargement. When they increased spontaneous release by using the complexin mutant, they found the opposite effect on bouton maturation. Interestingly, these structural synaptic changes were dependent on presynaptic Trio (a GEF) and Rac1 acting downstream of spontaneous release and directly modulating the actin cytoskeleton (Choi et al., 2014). Although it is still unclear how synapses distinguish between evoked and spontaneous release to modulate distinct pathways, studies in hippocampal neurons have shown each mode of release can activate distinct subsets of postsynaptic glutamate (NMDA and AMPA) receptors (Atasoy et al., 2008; Reese & Kavalali, 2016; Sara, Bal, Adachi, Monteggia, & Kavalali, 2011). Similar notions have also recently been reported in *Drosophila* synapses (Melom, Akbergenova, Gavornik, & Littleton, 2013; Peled, Newman, & Isacoff, 2014), raising the intriguing possibility that spontaneous and evoked release activate distinct postsynaptic molecular cascades by acting on spatially segregated receptors (Kavalali, 2015).

## 3 ROLE FOR SPONTANEOUS NEUROTRANSMISSION ON POSTSYNAPTIC DEVELOPMENT AND DENDRITIC ARBOR FORMATION

Evidence that spontaneous neurotransmitter release might be playing a role in the dendritic growth of excitatory neurons has been around since the 1990s, even though it was often not directly studied at the time. One of the first clues came from experiments examining the effects of the neurotrophin, brain‐derived neurotrophic factor (BDNF), on the growth of layer 4 cortical pyramidal neurons in cultured slices of ferret visual cortex (McAllister, Katz, & Lo, 1996). These are excitatory neurons with both an apical and basal dendritic arbor, and treatment with BDNF resulted in increased growth of both arbors. However, when the authors attempted to interfere with the effects of BDNF by blocking activity, some interesting distinctions emerged. In the apical dendrites, blocking action potential firing (with tetrodotoxin [TTX]), and hence evoked transmitter release, or glutamate receptors (with either CNQX to antagonise AMPA receptors, or APV to antagonise NMDA receptors) each reduced the effects of BDNF on growth. However, in basal dendrites, TTX alone had a minimal effect whereas glutamate receptor antagonists prevented the BDNF‐induced growth, suggesting that spontaneous (but not evoked) release of glutamate is needed for this BDNF plasticity. More directly, an *in vivo* study of tectal neuron development in *Xenopus* found that NMDA receptor blockade led to decreased dendritic growth, a reduced rate of new branch additions and impaired branch extension, while blocking evoked transmitter release with TTX had no effect (Rajan, Witte, & Cline, 1999), again implicating spontaneous release.

Excitatory, glutamatergic synapses exhibit a distinct morphology at the postsynaptic side in the form of dendritic protrusions, or spines. A key study specifically examining the role of spontaneous transmission in rodent hippocampal neurons found that it was critical for the maintenance of dendritic spines, especially in the context of deafferentation of spine inputs (McKinney, Capogna, Durr, Gahwiler, & Thompson, 1999). Although this was less of a true developmental effect, it did firmly point the finger at spontaneous glutamate release in the regulation of spine density, which could have obvious implications earlier in development. Further evidence that spontaneous transmission is important for excitatory synapse stability in mature neurons came from the demonstration that NMDA receptor dependent miniature currents help to keep homeostatic synaptic scaling in check during blockade of neuronal firing by restricting synthesis of certain AMPA receptor subunits (Sutton et al., 2006; see accompanying review on this topic in this issue). Interestingly, work at the *Drosophila* NMJ utilizing mutants of presynaptic function also suggested that the spontaneous release of glutamate could control postsynaptic glutamate receptor clustering (Saitoe, Schwarz, Umbach, Gundersen, & Kidokoro, 2001), which in mammals is strongly correlated with spine size (Matsuzaki et al., 2001; Zito, Scheuss, Knott, Hill, & Svoboda, 2009), although these findings are controversial (Featherstone, Rushton, & Broadie, 2002), possibly due to variations in the extent to which spontaneous release is impaired in different mutant flies (Featherstone & Broadie, 2002; Saitoe et al., 2002).

A recent study delved further into a possible role for spontaneous release early in development, given the very high levels of spontaneous vesicle cycling seen in more immature rodent neurons (Andreae et al., 2012). This found that spontaneously released glutamate from young axons could signal to distant NMDA receptors on developing dendrites. To dissect out the specific role of spontaneous release the authors were able to take advantage of a culture system where neuronal development was relatively synchronized, meaning that at specified early stages only spontaneous release was present, while at later stages evoked release was predominant. Blocking NMDA receptors early on, during a period where neurotransmitter release was exclusively spontaneous, resulted in a dramatic reduction in dendritic arbor complexity, while neither TTX (to block neuronal firing) nor NMDA blockade (at later stages) had any effect. Interfering with spontaneously released glutamate reduced dendritic complexity and also resulted in straighter dendrites with more symmetrical arbors (Figure 1C). This suggested that the released glutamate might be acting as a kind of dendritic branch guidance, or growth promoting, cue. Indeed, several instances of dendritic filopodia reaching out and targeting axonal boutons were observed, at sites where spontaneously cycling vesicles had been specifically labelled (Andreae & Burrone, 2015).

## 4 CONCLUSIONS AND CONTROVERSIES

In summary, there is now a significant body of evidence that spontaneous neurotransmitter release plays an important role in the development of neuronal connections and dendritic arbors. Understanding how synapses and circuits develop is increasingly relevant, as it becomes clear that many neurodevelopmental disorders, such as autism or intellectual disability, are likely to involve disruptions to these processes. Wiring the brain correctly early in development may be critical for allowing subsequent complex learning to take place. Indeed, a recent computational study that modelled the development of different forms of release found that early spontaneous release (with lower levels of evoked release) resulted in a homogenization of synaptic weights initially, which was critical to maintain an appropriate dynamic range of weights and might ‘prepare’ circuits for learning (Martens, Celikel, & Tiesinga, 2015). But many questions remain. Most vertebrate studies have been carried out using *in vitro* systems, and it will be important to validate these findings *in vivo*.

Also, the extent to which neurotransmitter release in general drives synaptic and neuronal development remains a controversial question. Indeed, a recent study examining hippocampal CA1 subfield pyramidal neurons from mice that lacked both spontaneous and evoked release (due to the absence of key SNARE proteins) found no profound changes (Sigler et al., 2017) while another study which used tetanus toxin to block release describe the loss of almost half of excitatory synapses on to the same neuronal type, with impaired dendritic arborizations (Sando et al., 2017). There is a great deal of evidence from the literature that using different approaches to modulating activity can result in different effects on synapse formation (Andreae & Burrone, 2014), and indeed, it is unclear how much of an impact tetanus toxin has on early spontaneous release (Andreae & Burrone, 2015; Choi et al., 2014; Shin et al., 2012; Verderio et al., 1999). Interestingly, the second study found very different effects on neurons in the CA3 region of the hippocampus, suggesting that even within the same microcircuit, the role of activity can be varied. This phenomenon has been elegantly described in the mouse retina where selective silencing of specific neurons had completely different impacts on synapse formation onto individual neuronal subtypes (Dunn & Wong, 2012; Kerschensteiner, Morgan, Parker, Lewis, & Wong, 2009; Morgan, Soto, Wong, & Kerschensteiner, 2011; Soto et al., 2012). Similarly, different interneuron subtypes in the developing mouse cortex are differentially affected by modifying activity (De Marco Garcia, Karayannis, & Fishell, 2011), which was dependent on input source acting through different NMDA subtypes (De Marco Garcia, Priya, Tuncdemir, Fishell, & Karayannis, 2015). In addition, competitive processes that arise from differences in neurotransmitter release between neurons may have a significant impact on connectivity, meaning that care is needed in interpreting studies that focus on the complete removal of release from all neurons. Approaches where release is selectively removed from a subset of neurons will allow a more in depth investigation as to how release affects connectivity.

Almost all the work investigating the role of spontaneous neurotransmission has focused on the development of glutamatergic synapses, but might similar mechanisms be employed at inhibitory, GABAergic synapses? GABAergic interneurons have a very different developmental programme in terms of regional origin, transcriptional regulators and migration patterns from excitatory neurons, and mature GABAergic synapses often show much higher levels of spontaneous release, but whether this is seen during development and the role it might play is unknown. The nervous system is extraordinarily diverse, not just in terms of neuronal cell types or morphology, but at multiple levels including functional patterns of behaviour, plasticity rules and mechanisms, and circuit structure and function. How different types of neurotransmitter release regulate the formation of different synaptic connections and circuits will be a critical question for the future.

## CONFLICT OF INTEREST

None

## AUTHOR CONTRIBUTIONS

All authors had full access to all the data in the study and take responsibility for the integrity of the data and the accuracy of the data analysis. Conceptualization, Methodology, Investigation, Formal Analysis, Resources, Visualization, Supervision, Funding Acquisition, Writing, Review & Editing: LCA and JB.
